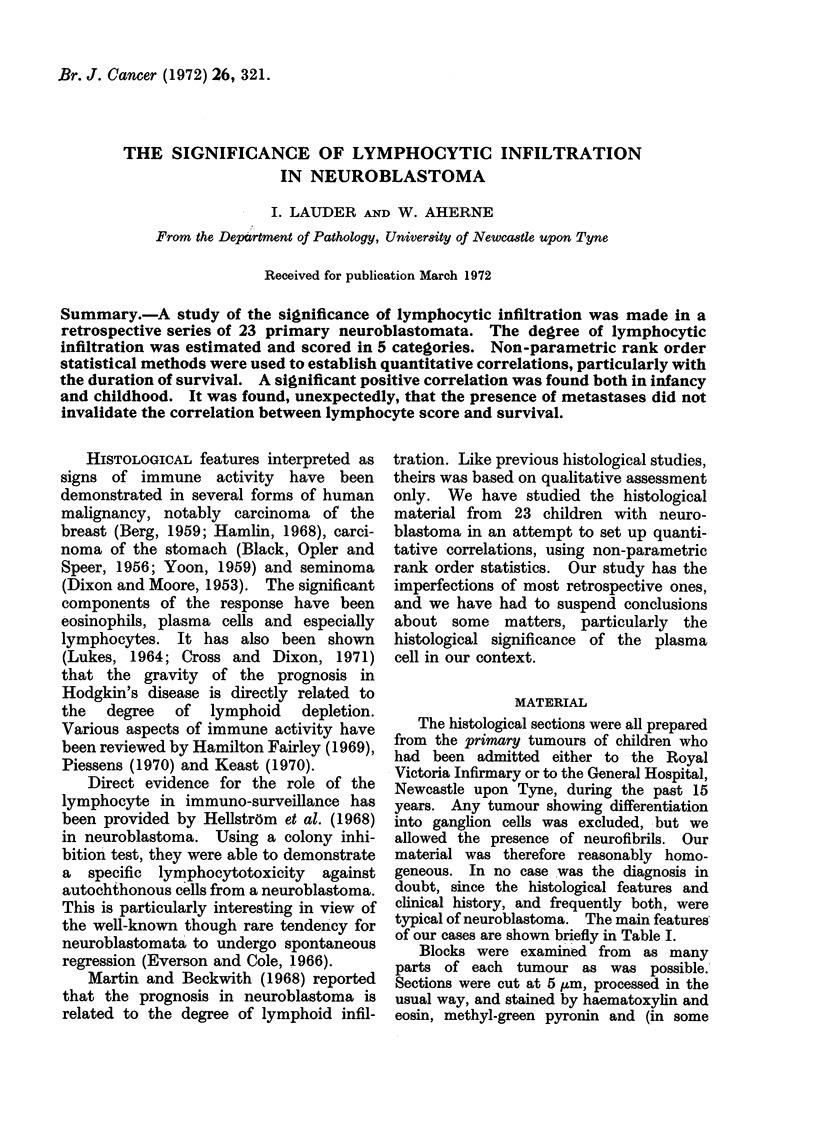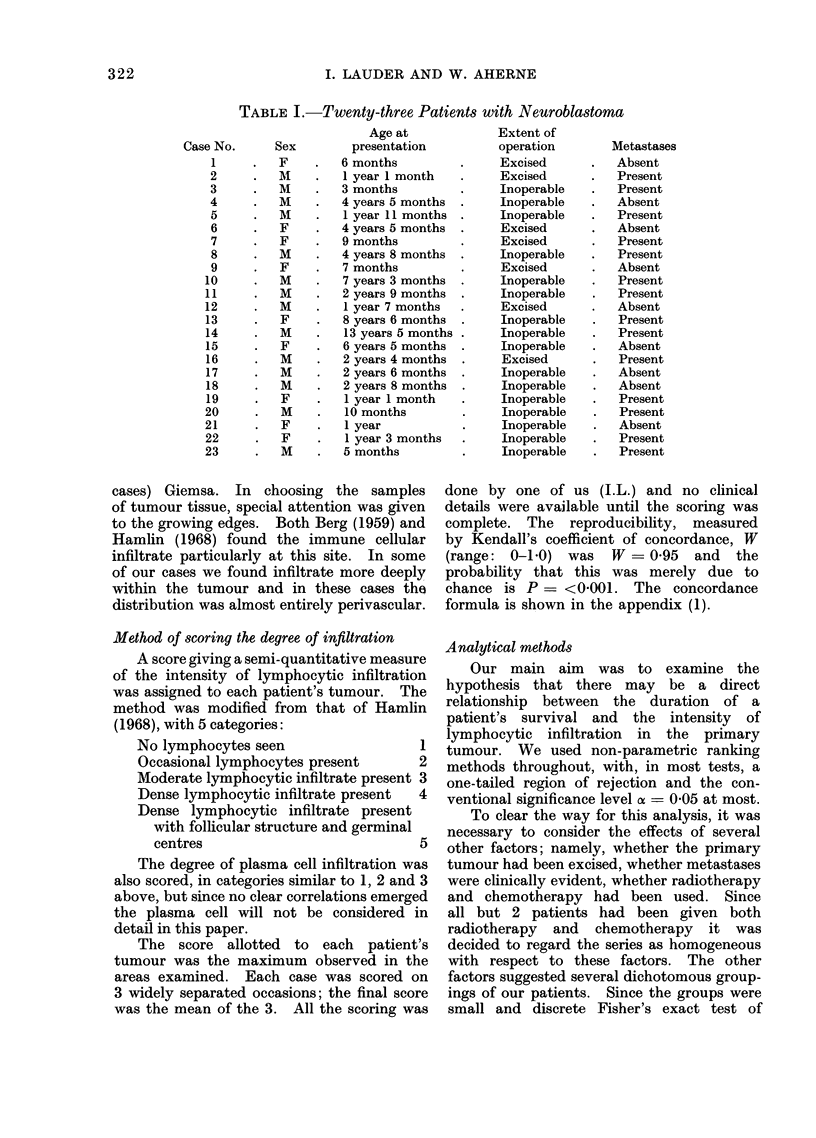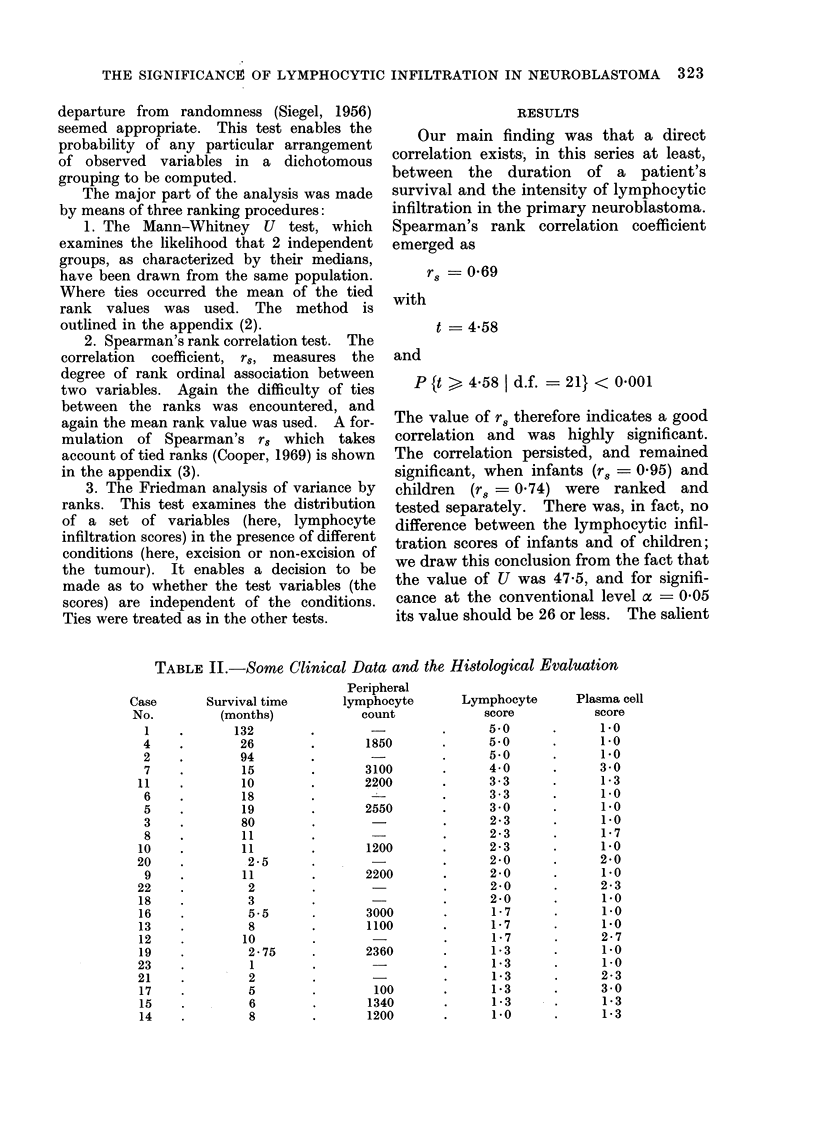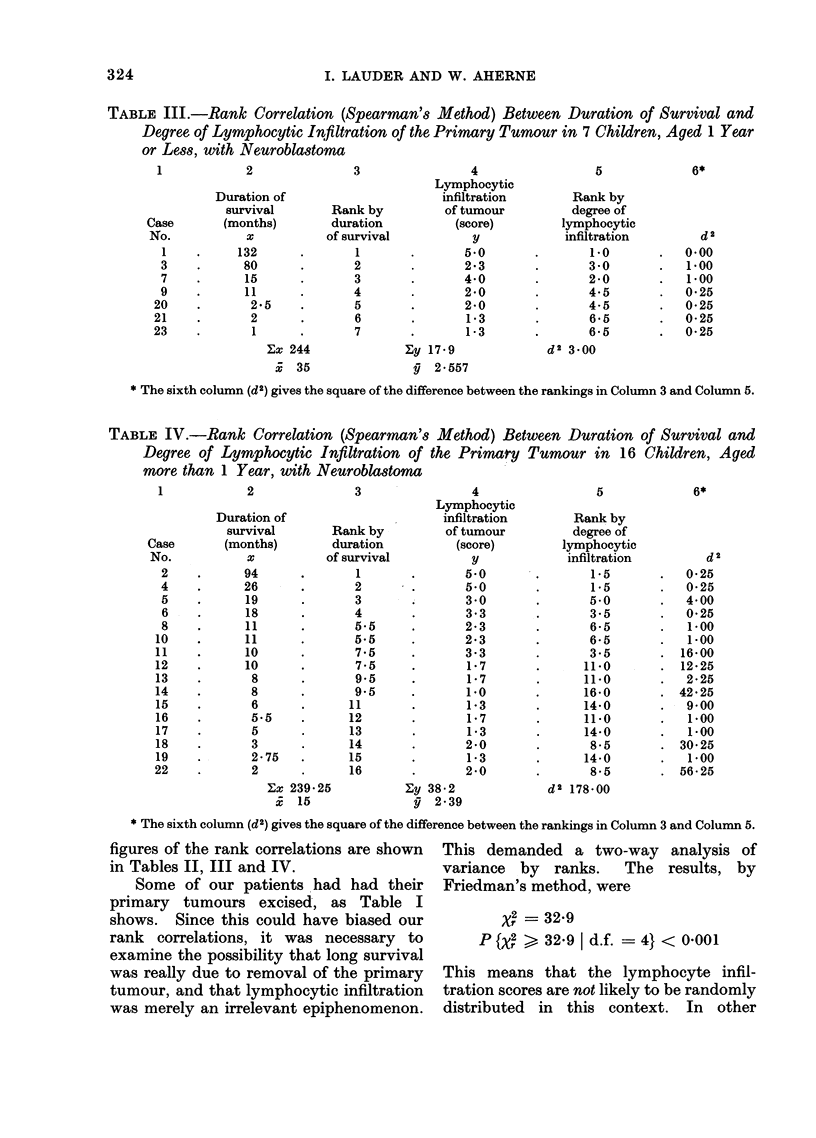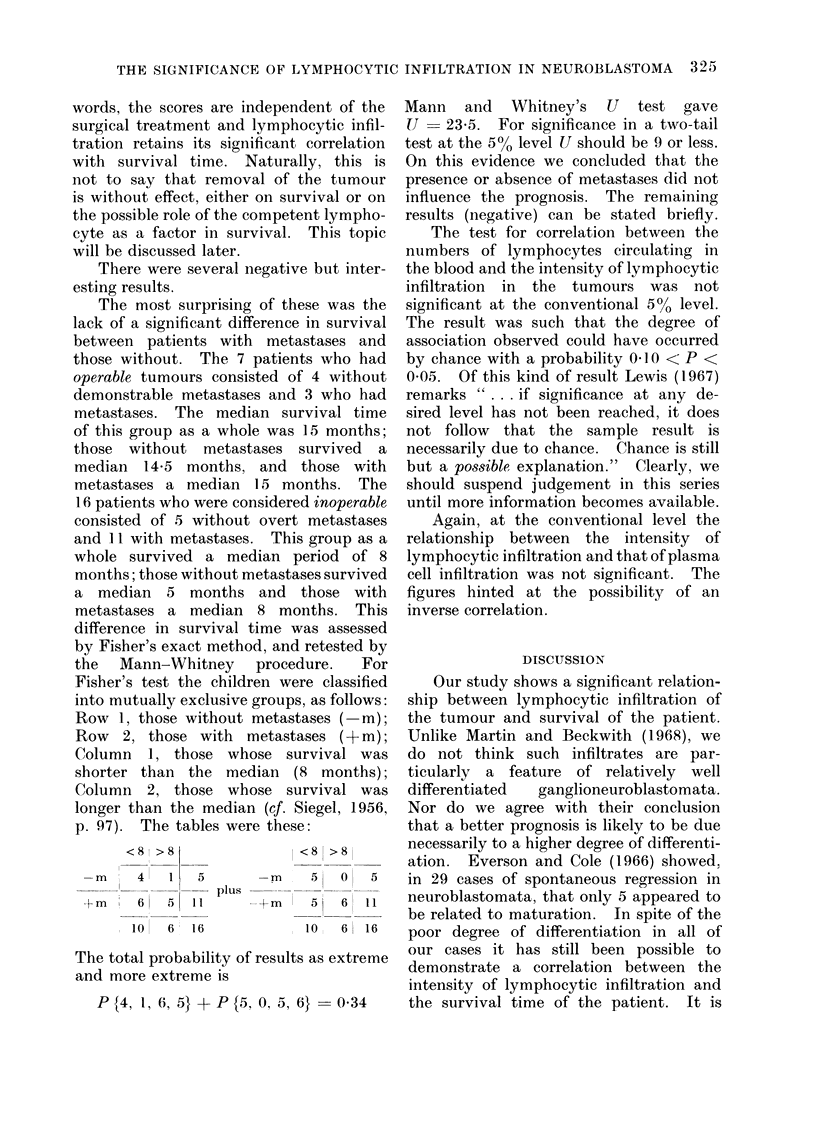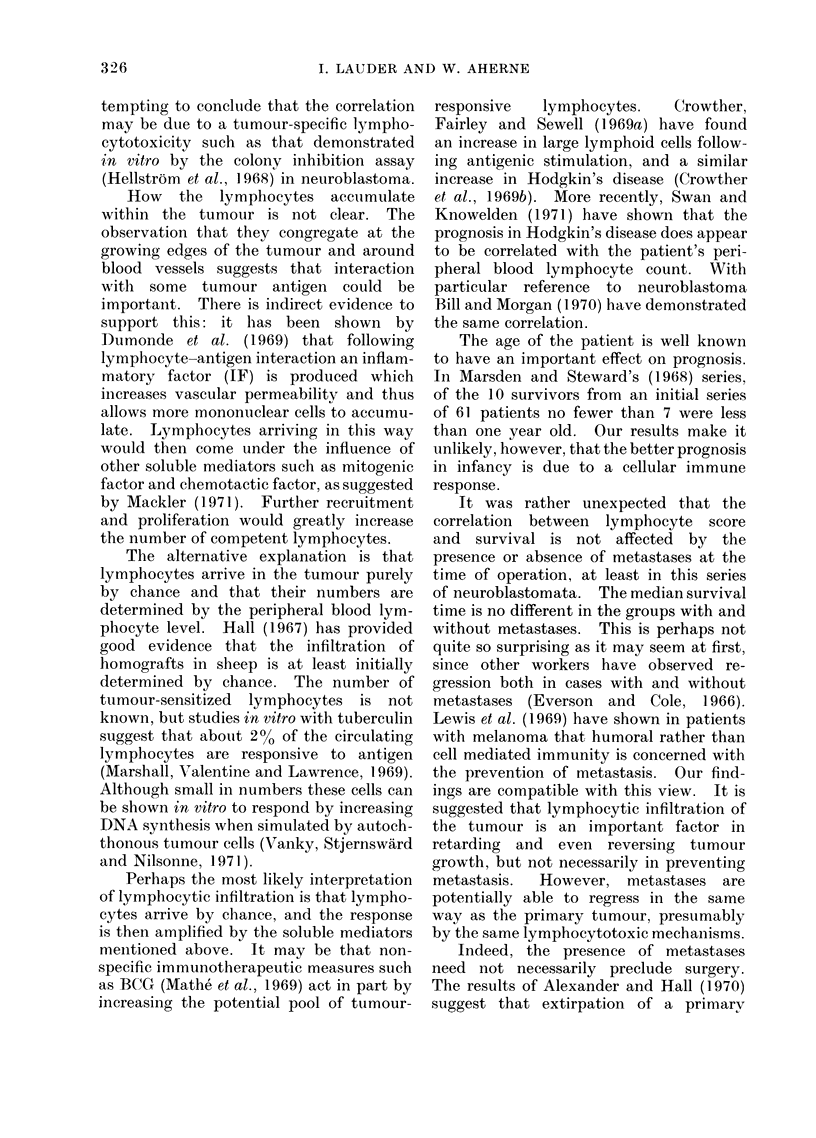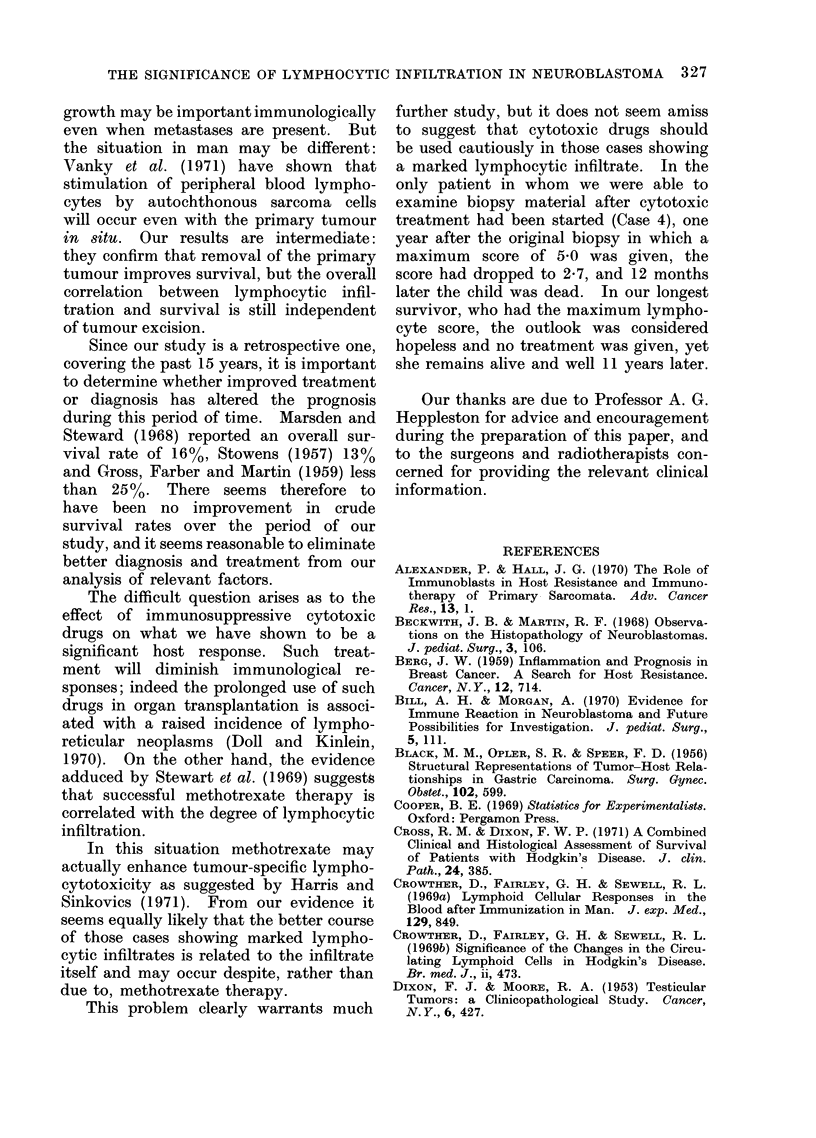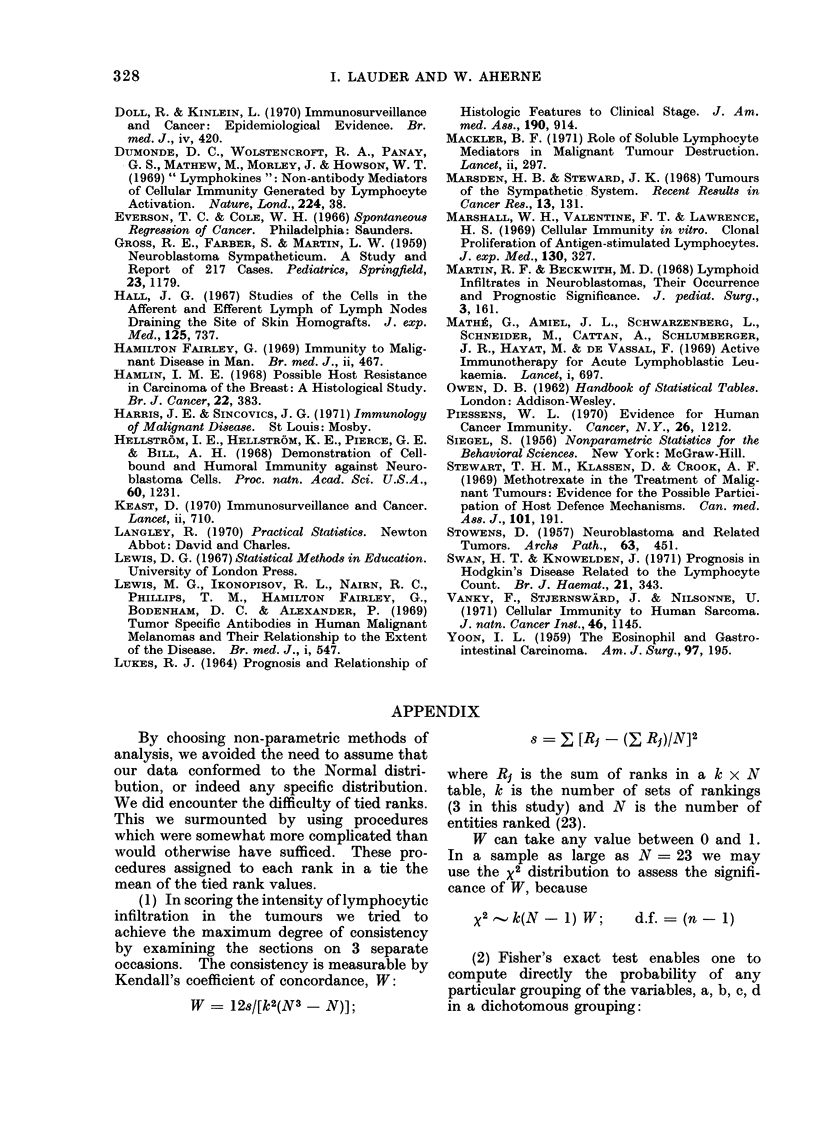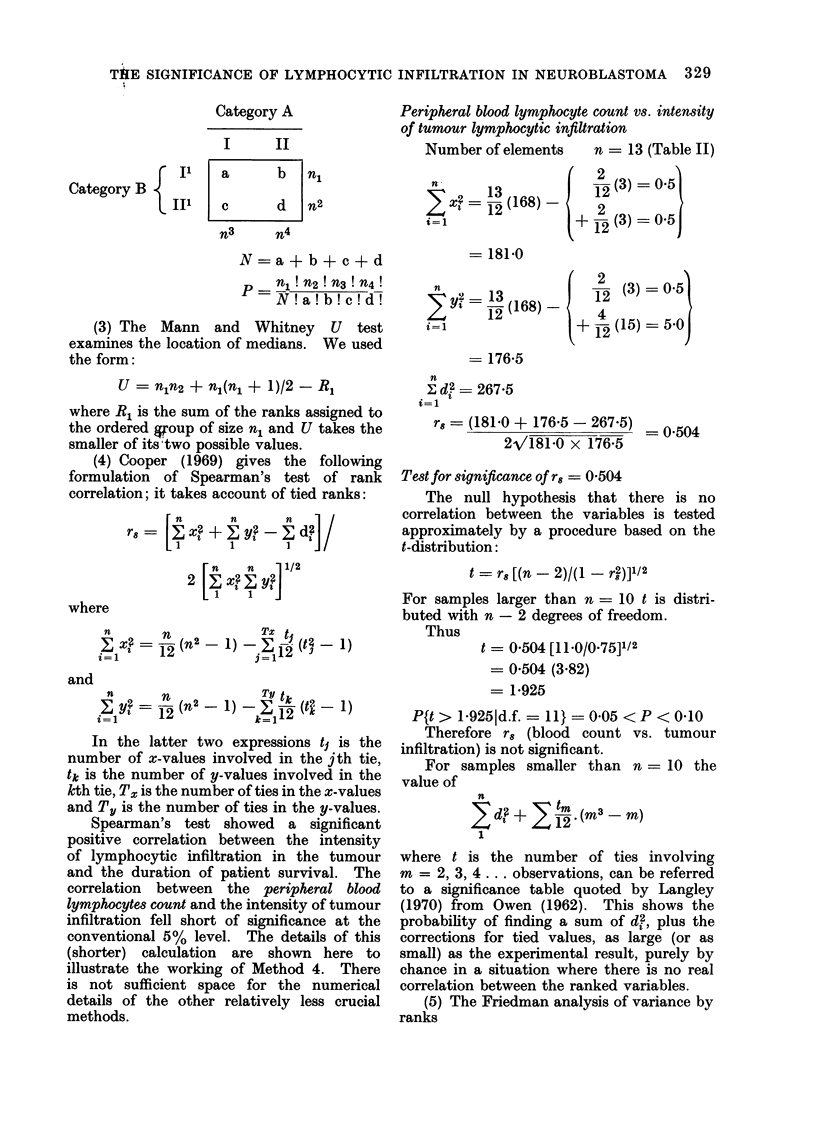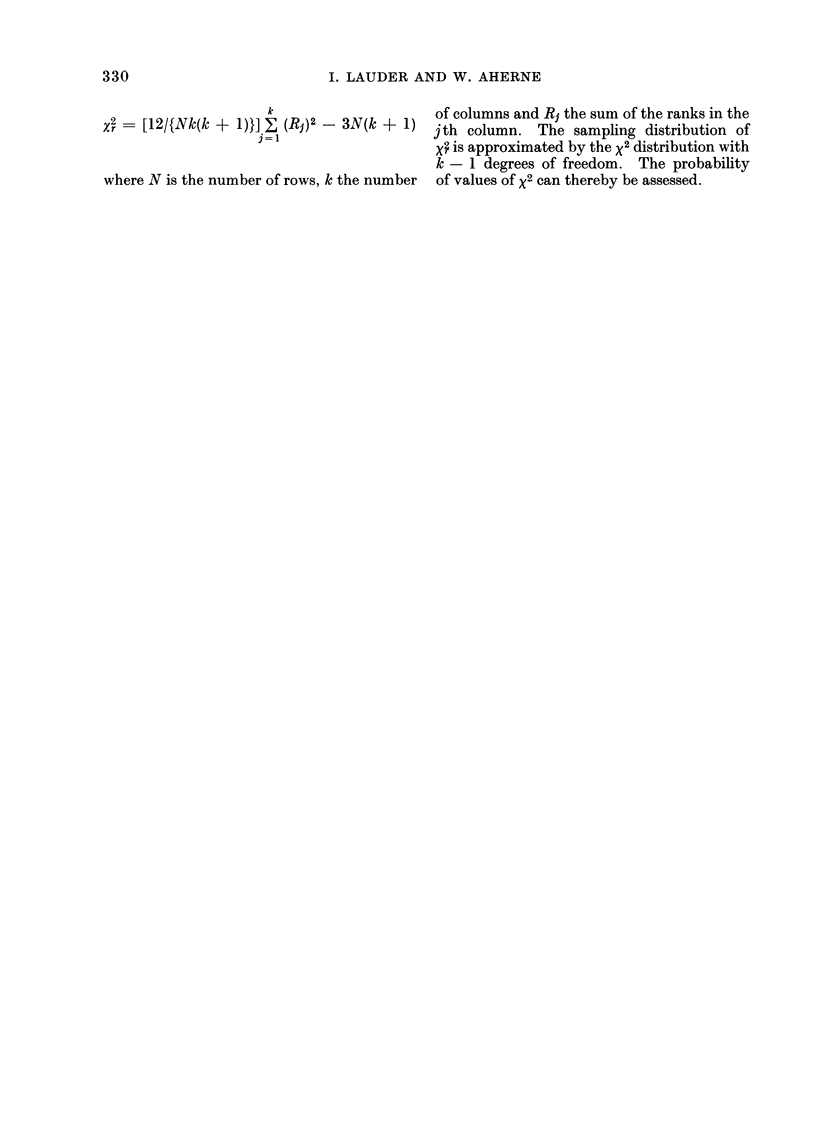# The Significance of Lymphocytic Infiltration in Neuroblastoma

**DOI:** 10.1038/bjc.1972.43

**Published:** 1972-08

**Authors:** I. Lauder, W. Aherne

## Abstract

A study of the significance of lymphocytic infiltration was made in a retrospective series of 23 primary neuroblastomata. The degree of lymphocytic infiltration was estimated and scored in 5 categories. Non-parametric rank order statistical methods were used to establish quantitative correlations, particularly with the duration of survival. A significant positive correlation was found both in infancy and childhood. It was found, unexpectedly, that the presence of metastases did not invalidate the correlation between lymphocyte score and survival.


*Br. J. Cancer* (1972) **26**, 321.

### THE SIGNIFICANCE OF LYMPHOCYTIC INFILTRATION IN NEUROBLASTOMA

I. LAUDER AND W. AHERNE

*From the Department of Pathology, University of Newcastle upon Tyne*

Received for publication March 1972

**Summary.—A study of the significance of lymphocytic infiltration was made in a retrospective series of 23 primary neuroblastomata. The degree of lymphocytic infiltration was estimated and scored in 5 categories. Non-parametric rank order statistical methods were used to establish quantitative correlations, particularly with the duration of survival. A significant positive correlation was found both in infancy and childhood. It was found, unexpectedly, that the presence of metastases did not invalidate the correlation between lymphocyte score and survival.**

HISTOLOGICAL features interpreted as signs of immune activity have been demonstrated in several forms of human malignancy, notably carcinoma of the breast (Berg, 1959; Hamlin, 1968), carcinoma of the stomach (Black, Opler and Speer, 1956; Yoon, 1959) and seminoma (Dixon and Moore, 1953). The significant components of the response have been eosinophils, plasma cells and especially lymphocytes. It has also been shown (Lukes, 1964; Cross and Dixon, 1971) that the gravity of the prognosis in Hodgkin's disease is directly related to the degree of lymphoid depletion. Various aspects of immune activity have been reviewed by Hamilton Fairley (1969), Piessens (1970) and Keast (1970).

Direct evidence for the role of the lymphocyte in immuno-surveillance has been provided by Hellström *et al.* (1968) in neuroblastoma. Using a colony inhibition test, they were able to demonstrate a specific lymphocytotoxicity against autochthonous cells from a neuroblastoma. This is particularly interesting in view of the well-known though rare tendency for neuroblastomata to undergo spontaneous regression (Everson and Cole, 1966).

Martin and Beckwith (1968) reported that the prognosis in neuroblastoma is related to the degree of lymphoid infiltration. Like previous histological studies, theirs was based on qualitative assessment only. We have studied the histological material from 23 children with neuroblastoma in an attempt to set up quantitative correlations, using non-parametric rank order statistics. Our study has the imperfections of most retrospective ones, and we have had to suspend conclusions about some matters, particularly the histological significance of the plasma cell in our context.

### MATERIAL

The histological sections were all prepared from the *primary* tumours of children who had been admitted either to the Royal Victoria Infirmary or to the General Hospital, Newcastle upon Tyne, during the past 15 years. Any tumour showing differentiation into ganglion cells was excluded, but we allowed the presence of neurofibrils. Our material was therefore reasonably homogeneous. In no case was the diagnosis in doubt, since the histological features and clinical history, and frequently both, were typical of neuroblastoma. The main features of our cases are shown briefly in Table I.

Blocks were examined from as many parts of each tumour as was possible. Sections were cut at 5 μm, processed in the usual way, and stained by haematoxylin and eosin, methyl-green pyronin and (in some

TABLE I.—*Twenty-three Patients with Neuroblastoma*

| Case No. | Sex | Age at presentation | Extent of operation | Metastases |
|---|---|---|---|---|
| 1 | F | 6 months | Excised | Absent |
| 2 | M | 1 year 1 month | Excised | Present |
| 3 | M | 3 months | Inoperable | Present |
| 4 | M | 4 years 5 months | Inoperable | Absent |
| 5 | M | 1 year 11 months | Inoperable | Present |
| 6 | F | 4 years 5 months | Excised | Absent |
| 7 | F | 9 months | Excised | Present |
| 8 | M | 4 years 8 months | Inoperable | Present |
| 9 | F | 7 months | Excised | Absent |
| 10 | M | 7 years 3 months | Inoperable | Present |
| 11 | M | 2 years 9 months | Inoperable | Present |
| 12 | M | 1 year 7 months | Excised | Absent |
| 13 | F | 8 years 6 months | Inoperable | Present |
| 14 | M | 13 years 5 months | Inoperable | Present |
| 15 | F | 6 years 5 months | Inoperable | Absent |
| 16 | M | 2 years 4 months | Excised | Present |
| 17 | M | 2 years 6 months | Inoperable | Absent |
| 18 | M | 2 years 8 months | Inoperable | Absent |
| 19 | F | 1 year 1 month | Inoperable | Present |
| 20 | M | 10 months | Inoperable | Present |
| 21 | F | 1 year | Inoperable | Absent |
| 22 | F | 1 year 3 months | Inoperable | Present |
| 23 | M | 5 months | Inoperable | Present |

cases) Giemsa. In choosing the samples of tumour tissue, special attention was given to the growing edges. Both Berg (1959) and Hamlin (1968) found the immune cellular infiltrate particularly at this site. In some of our cases we found infiltrate more deeply within the tumour and in these cases the distribution was almost entirely perivascular.

*Method of scoring the degree of infiltration*

A score giving a semi-quantitative measure of the intensity of lymphocytic infiltration was assigned to each patient's tumour. The method was modified from that of Hamlin (1968), with 5 categories:

| | |
|---|---|
| No lymphocytes seen | 1 |
| Occasional lymphocytes present | 2 |
| Moderate lymphocytic infiltrate present | 3 |
| Dense lymphocytic infiltrate present | 4 |
| Dense lymphocytic infiltrate present with follicular structure and germinal centres | 5 |

The degree of plasma cell infiltration was also scored, in categories similar to 1, 2 and 3 above, but since no clear correlations emerged the plasma cell will not be considered in detail in this paper.

The score allotted to each patient's tumour was the maximum observed in the areas examined. Each case was scored on 3 widely separated occasions; the final score was the mean of the 3. All the scoring was done by one of us (I.L.) and no clinical details were available until the scoring was complete. The reproducibility, measured by Kendall's coefficient of concordance, $W$ (range: 0–1·0) was $W = 0{\cdot}95$ and the probability that this was merely due to chance is $P = <0{\cdot}001$. The concordance formula is shown in the appendix (1).

*Analytical methods*

Our main aim was to examine the hypothesis that there may be a direct relationship between the duration of a patient's survival and the intensity of lymphocytic infiltration in the primary tumour. We used non-parametric ranking methods throughout, with, in most tests, a one-tailed region of rejection and the conventional significance level $\alpha = 0{\cdot}05$ at most.

To clear the way for this analysis, it was necessary to consider the effects of several other factors; namely, whether the primary tumour had been excised, whether metastases were clinically evident, whether radiotherapy and chemotherapy had been used. Since all but 2 patients had been given both radiotherapy and chemotherapy it was decided to regard the series as homogeneous with respect to these factors. The other factors suggested several dichotomous groupings of our patients. Since the groups were small and discrete Fisher's exact test of departure from randomness (Siegel, 1956) seemed appropriate. This test enables the probability of any particular arrangement of observed variables in a dichotomous grouping to be computed.

The major part of the analysis was made by means of three ranking procedures:

1. The Mann–Whitney $U$ test, which examines the likelihood that 2 independent groups, as characterized by their medians, have been drawn from the same population. Where ties occurred the mean of the tied rank values was used. The method is outlined in the appendix (2).

2. Spearman's rank correlation test. The correlation coefficient, $r_s$, measures the degree of rank ordinal association between two variables. Again the difficulty of ties between the ranks was encountered, and again the mean rank value was used. A formulation of Spearman's $r_s$ which takes account of tied ranks (Cooper, 1969) is shown in the appendix (3).

3. The Friedman analysis of variance by ranks. This test examines the distribution of a set of variables (here, lymphocyte infiltration scores) in the presence of different conditions (here, excision or non-excision of the tumour). It enables a decision to be made as to whether the test variables (the scores) are independent of the conditions. Ties were treated as in the other tests.

### RESULTS

Our main finding was that a direct correlation exists, in this series at least, between the duration of a patient's survival and the intensity of lymphocytic infiltration in the primary neuroblastoma. Spearman's rank correlation coefficient emerged as

$$r_s = 0{\cdot}69$$

with

$$t = 4{\cdot}58$$

and

$$P\{t \geqslant 4{\cdot}58 \mid \text{d.f.} = 21\} < 0{\cdot}001$$

The value of $r_s$ therefore indicates a good correlation and was highly significant. The correlation persisted, and remained significant, when infants ($r_s = 0{\cdot}95$) and children ($r_s = 0{\cdot}74$) were ranked and tested separately. There was, in fact, no difference between the lymphocytic infiltration scores of infants and of children; we draw this conclusion from the fact that the value of $U$ was 47·5, and for significance at the conventional level $\alpha = 0{\cdot}05$ its value should be 26 or less. The salient

TABLE II.—*Some Clinical Data and the Histological Evaluation*

| Case No. | Survival time (months) | Peripheral lymphocyte count | Lymphocyte score | Plasma cell score |
|---|---|---|---|---|
| 1 | 132 | — | 5·0 | 1·0 |
| 4 | 26 | 1850 | 5·0 | 1·0 |
| 2 | 94 | — | 5·0 | 1·0 |
| 7 | 15 | 3100 | 4·0 | 3·0 |
| 11 | 10 | 2200 | 3·3 | 1·3 |
| 6 | 18 | — | 3·3 | 1·0 |
| 5 | 19 | 2550 | 3·0 | 1·0 |
| 3 | 80 | — | 2·3 | 1·0 |
| 8 | 11 | — | 2·3 | 1·7 |
| 10 | 11 | 1200 | 2·3 | 1·0 |
| 20 | 2·5 | — | 2·0 | 2·0 |
| 9 | 11 | 2200 | 2·0 | 1·0 |
| 22 | 2 | — | 2·0 | 2·3 |
| 18 | 3 | — | 2·0 | 1·0 |
| 16 | 5·5 | 3000 | 1·7 | 1·0 |
| 13 | 8 | 1100 | 1·7 | 1·0 |
| 12 | 10 | — | 1·7 | 2·7 |
| 19 | 2·75 | 2360 | 1·3 | 1·0 |
| 23 | 1 | — | 1·3 | 1·0 |
| 21 | 2 | — | 1·3 | 2·3 |
| 17 | 5 | 100 | 1·3 | 3·0 |
| 15 | 6 | 1340 | 1·3 | 1·3 |
| 14 | 8 | 1200 | 1·0 | 1·3 |

TABLE III.—*Rank Correlation (Spearman's Method) Between Duration of Survival and Degree of Lymphocytic Infiltration of the Primary Tumour in 7 Children, Aged 1 Year or Less, with Neuroblastoma*

| 1<br>Case No. | 2<br>Duration of survival (months)<br>$x$ | 3<br>Rank by duration of survival | 4<br>Lymphocytic infiltration of tumour (score)<br>$y$ | 5<br>Rank by degree of lymphocytic infiltration | 6*<br>$d^2$ |
|---|---|---|---|---|---|
| 1 | 132 | 1 | 5·0 | 1·0 | 0·00 |
| 3 | 80 | 2 | 2·3 | 3·0 | 1·00 |
| 7 | 15 | 3 | 4·0 | 2·0 | 1·00 |
| 9 | 11 | 4 | 2·0 | 4·5 | 0·25 |
| 20 | 2·5 | 5 | 2·0 | 4·5 | 0·25 |
| 21 | 2 | 6 | 1·3 | 6·5 | 0·25 |
| 23 | 1 | 7 | 1·3 | 6·5 | 0·25 |
| | $\Sigma x$ 244 | | $\Sigma y$ 17·9 | $d^2$ 3·00 | |
| | $\bar{x}$ 35 | | $\bar{y}$ 2·557 | | |

* The sixth column ($d^2$) gives the square of the difference between the rankings in Column 3 and Column 5.

TABLE IV.—*Rank Correlation (Spearman's Method) Between Duration of Survival and Degree of Lymphocytic Infiltration of the Primary Tumour in 16 Children, Aged more than 1 Year, with Neuroblastoma*

| 1<br>Case No. | 2<br>Duration of survival (months)<br>$x$ | 3<br>Rank by duration of survival | 4<br>Lymphocytic infiltration of tumour (score)<br>$y$ | 5<br>Rank by degree of lymphocytic infiltration | 6*<br>$d^2$ |
|---|---|---|---|---|---|
| 2 | 94 | 1 | 5·0 | 1·5 | 0·25 |
| 4 | 26 | 2 | 5·0 | 1·5 | 0·25 |
| 5 | 19 | 3 | 3·0 | 5·0 | 4·00 |
| 6 | 18 | 4 | 3·3 | 3·5 | 0·25 |
| 8 | 11 | 5·5 | 2·3 | 6·5 | 1·00 |
| 10 | 11 | 5·5 | 2·3 | 6·5 | 1·00 |
| 11 | 10 | 7·5 | 3·3 | 3·5 | 16·00 |
| 12 | 10 | 7·5 | 1·7 | 11·0 | 12·25 |
| 13 | 8 | 9·5 | 1·7 | 11·0 | 2·25 |
| 14 | 8 | 9·5 | 1·0 | 16·0 | 42·25 |
| 15 | 6 | 11 | 1·3 | 14·0 | 9·00 |
| 16 | 5·5 | 12 | 1·7 | 11·0 | 1·00 |
| 17 | 5 | 13 | 1·3 | 14·0 | 1·00 |
| 18 | 3 | 14 | 2·0 | 8·5 | 30·25 |
| 19 | 2·75 | 15 | 1·3 | 14·0 | 1·00 |
| 22 | 2 | 16 | 2·0 | 8·5 | 56·25 |
| | $\Sigma x$ 239·25 | | $\Sigma y$ 38·2 | $d^2$ 178·00 | |
| | $\bar{x}$ 15 | | $\bar{y}$ 2·39 | | |

* The sixth column ($d^2$) gives the square of the difference between the rankings in Column 3 and Column 5.

figures of the rank correlations are shown in Tables II, III and IV.

Some of our patients had had their primary tumours excised, as Table I shows. Since this could have biased our rank correlations, it was necessary to examine the possibility that long survival was really due to removal of the primary tumour, and that lymphocytic infiltration was merely an irrelevant epiphenomenon. This demanded a two-way analysis of variance by ranks. The results, by Friedman's method, were

$$\chi_r^2 = 32{\cdot}9$$

$$P\{\chi_r^2 \geqslant 32{\cdot}9 \mid \text{d.f.} = 4\} < 0{\cdot}001$$

This means that the lymphocyte infiltration scores are *not* likely to be randomly distributed in this context. In other words, the scores are independent of the surgical treatment and lymphocytic infiltration retains its significant correlation with survival time. Naturally, this is not to say that removal of the tumour is without effect, either on survival or on the possible role of the competent lymphocyte as a factor in survival. This topic will be discussed later.

There were several negative but interesting results.

The most surprising of these was the lack of a significant difference in survival between patients with metastases and those without. The 7 patients who had *operable* tumours consisted of 4 without demonstrable metastases and 3 who had metastases. The median survival time of this group as a whole was 15 months; those without metastases survived a median 14·5 months, and those with metastases a median 15 months. The 16 patients who were considered *inoperable* consisted of 5 without overt metastases and 11 with metastases. This group as a whole survived a median period of 8 months; those without metastases survived a median 5 months and those with metastases a median 8 months. This difference in survival time was assessed by Fisher's exact method, and retested by the Mann–Whitney procedure. For Fisher's test the children were classified into mutually exclusive groups, as follows: Row 1, those without metastases (−m); Row 2, those with metastases (+m); Column 1, those whose survival was shorter than the median (8 months); Column 2, those whose survival was longer than the median (*cf.* Siegel, 1956, p. 97). The tables were these:

| | <8 | >8 | |
|---|---|---|---|
| −m | 4 | 1 | 5 |
| +m | 6 | 5 | 11 |
| | 10 | 6 | 16 |

plus

| | <8 | >8 | |
|---|---|---|---|
| −m | 5 | 0 | 5 |
| +m | 5 | 6 | 11 |
| | 10 | 6 | 16 |

The total probability of results as extreme and more extreme is

$$P\{4, 1, 6, 5\} + P\{5, 0, 5, 6\} = 0{\cdot}34$$

Mann and Whitney's $U$ test gave $U = 23{\cdot}5$. For significance in a two-tail test at the 5% level $U$ should be 9 or less. On this evidence we concluded that the presence or absence of metastases did not influence the prognosis. The remaining results (negative) can be stated briefly.

The test for correlation between the numbers of lymphocytes circulating in the blood and the intensity of lymphocytic infiltration in the tumours was not significant at the conventional 5% level. The result was such that the degree of association observed could have occurred by chance with a probability $0{\cdot}10 < P < 0{\cdot}05$. Of this kind of result Lewis (1967) remarks " . . . if significance at any desired level has not been reached, it does not follow that the sample result is necessarily due to chance. Chance is still but a *possible* explanation." Clearly, we should suspend judgement in this series until more information becomes available.

Again, at the conventional level the relationship between the intensity of lymphocytic infiltration and that of plasma cell infiltration was not significant. The figures hinted at the possibility of an inverse correlation.

### DISCUSSION

Our study shows a significant relationship between lymphocytic infiltration of the tumour and survival of the patient. Unlike Martin and Beckwith (1968), we do not think such infiltrates are particularly a feature of relatively well differentiated ganglioneuroblastomata. Nor do we agree with their conclusion that a better prognosis is likely to be due necessarily to a higher degree of differentiation. Everson and Cole (1966) showed, in 29 cases of spontaneous regression in neuroblastomata, that only 5 appeared to be related to maturation. In spite of the poor degree of differentiation in all of our cases it has still been possible to demonstrate a correlation between the intensity of lymphocytic infiltration and the survival time of the patient. It is tempting to conclude that the correlation may be due to a tumour-specific lymphocytotoxicity such as that demonstrated *in vitro* by the colony inhibition assay (Hellström *et al.*, 1968) in neuroblastoma.

How the lymphocytes accumulate within the tumour is not clear. The observation that they congregate at the growing edges of the tumour and around blood vessels suggests that interaction with some tumour antigen could be important. There is indirect evidence to support this: it has been shown by Dumonde *et al.* (1969) that following lymphocyte–antigen interaction an inflammatory factor (IF) is produced which increases vascular permeability and thus allows more mononuclear cells to accumulate. Lymphocytes arriving in this way would then come under the influence of other soluble mediators such as mitogenic factor and chemotactic factor, as suggested by Mackler (1971). Further recruitment and proliferation would greatly increase the number of competent lymphocytes.

The alternative explanation is that lymphocytes arrive in the tumour purely by chance and that their numbers are determined by the peripheral blood lymphocyte level. Hall (1967) has provided good evidence that the infiltration of homografts in sheep is at least initially determined by chance. The number of tumour-sensitized lymphocytes is not known, but studies *in vitro* with tuberculin suggest that about 2% of the circulating lymphocytes are responsive to antigen (Marshall, Valentine and Lawrence, 1969). Although small in numbers these cells can be shown *in vitro* to respond by increasing DNA synthesis when simulated by autochthonous tumour cells (Vanky, Stjernswärd and Nilsonne, 1971).

Perhaps the most likely interpretation of lymphocytic infiltration is that lymphocytes arrive by chance, and the response is then amplified by the soluble mediators mentioned above. It may be that non-specific immunotherapeutic measures such as BCG (Mathé *et al.*, 1969) act in part by increasing the potential pool of tumour-responsive lymphocytes. Crowther, Fairley and Sewell (1969*a*) have found an increase in large lymphoid cells following antigenic stimulation, and a similar increase in Hodgkin's disease (Crowther *et al.*, 1969*b*). More recently, Swan and Knowelden (1971) have shown that the prognosis in Hodgkin's disease does appear to be correlated with the patient's peripheral blood lymphocyte count. With particular reference to neuroblastoma Bill and Morgan (1970) have demonstrated the same correlation.

The age of the patient is well known to have an important effect on prognosis. In Marsden and Steward's (1968) series, of the 10 survivors from an initial series of 61 patients no fewer than 7 were less than one year old. Our results make it unlikely, however, that the better prognosis in infancy is due to a cellular immune response.

It was rather unexpected that the correlation between lymphocyte score and survival is not affected by the presence or absence of metastases at the time of operation, at least in this series of neuroblastomata. The median survival time is no different in the groups with and without metastases. This is perhaps not quite so surprising as it may seem at first, since other workers have observed regression both in cases with and without metastases (Everson and Cole, 1966). Lewis *et al.* (1969) have shown in patients with melanoma that humoral rather than cell mediated immunity is concerned with the prevention of metastasis. Our findings are compatible with this view. It is suggested that lymphocytic infiltration of the tumour is an important factor in retarding and even reversing tumour growth, but not necessarily in preventing metastasis. However, metastases are potentially able to regress in the same way as the primary tumour, presumably by the same lymphocytotoxic mechanisms.

Indeed, the presence of metastases need not necessarily preclude surgery. The results of Alexander and Hall (1970) suggest that extirpation of a primary growth may be important immunologically even when metastases are present. But the situation in man may be different: Vanky *et al.* (1971) have shown that stimulation of peripheral blood lymphocytes by autochthonous sarcoma cells will occur even with the primary tumour *in situ*. Our results are intermediate: they confirm that removal of the primary tumour improves survival, but the overall correlation between lymphocytic infiltration and survival is still independent of tumour excision.

Since our study is a retrospective one, covering the past 15 years, it is important to determine whether improved treatment or diagnosis has altered the prognosis during this period of time. Marsden and Steward (1968) reported an overall survival rate of 16%, Stowens (1957) 13% and Gross, Farber and Martin (1959) less than 25%. There seems therefore to have been no improvement in crude survival rates over the period of our study, and it seems reasonable to eliminate better diagnosis and treatment from our analysis of relevant factors.

The difficult question arises as to the effect of immunosuppressive cytotoxic drugs on what we have shown to be a significant host response. Such treatment will diminish immunological responses; indeed the prolonged use of such drugs in organ transplantation is associated with a raised incidence of lymphoreticular neoplasms (Doll and Kinlein, 1970). On the other hand, the evidence adduced by Stewart *et al.* (1969) suggests that successful methotrexate therapy is correlated with the degree of lymphocytic infiltration.

In this situation methotrexate may actually enhance tumour-specific lymphocytotoxicity as suggested by Harris and Sinkovics (1971). From our evidence it seems equally likely that the better course of those cases showing marked lymphocytic infiltrates is related to the infiltrate itself and may occur despite, rather than due to, methotrexate therapy.

This problem clearly warrants much further study, but it does not seem amiss to suggest that cytotoxic drugs should be used cautiously in those cases showing a marked lymphocytic infiltrate. In the only patient in whom we were able to examine biopsy material after cytotoxic treatment had been started (Case 4), one year after the original biopsy in which a maximum score of 5·0 was given, the score had dropped to 2·7, and 12 months later the child was dead. In our longest survivor, who had the maximum lymphocyte score, the outlook was considered hopeless and no treatment was given, yet she remains alive and well 11 years later.

Our thanks are due to Professor A. G. Heppleston for advice and encouragement during the preparation of this paper, and to the surgeons and radiotherapists concerned for providing the relevant clinical information.

REFERENCES

Alexander, P. & Hall, J. G. (1970) The Role of Immunoblasts in Host Resistance and Immunotherapy of Primary Sarcomata. *Adv. Cancer Res.*, **13**, 1.

Beckwith, J. B. & Martin, R. F. (1968) Observations on the Histopathology of Neuroblastomas. *J. pediat. Surg.*, **3**, 106.

Berg, J. W. (1959) Inflammation and Prognosis in Breast Cancer. A Search for Host Resistance. *Cancer, N.Y.*, **12**, 714.

Bill, A. H. & Morgan, A. (1970) Evidence for Immune Reaction in Neuroblastoma and Future Possibilities for Investigation. *J. pediat. Surg.*, **5**, 111.

Black, M. M., Opler, S. R. & Speer, F. D. (1956) Structural Representations of Tumor–Host Relationships in Gastric Carcinoma. *Surg. Gynec. Obstet.*, **102**, 599.

Cooper, B. E. (1969) *Statistics for Experimentalists.* Oxford: Pergamon Press.

Cross, R. M. & Dixon, F. W. P. (1971) A Combined Clinical and Histological Assessment of Survival of Patients with Hodgkin's Disease. *J. clin. Path.*, **24**, 385.

Crowther, D., Fairley, G. H. & Sewell, R. L. (1969*a*) Lymphoid Cellular Responses in the Blood after Immunization in Man. *J. exp. Med.*, **129**, 849.

Crowther, D., Fairley, G. H. & Sewell, R. L. (1969*b*) Significance of the Changes in the Circulating Lymphoid Cells in Hodgkin's Disease. *Br. med. J.*, ii, 473.

Dixon, F. J. & Moore, R. A. (1953) Testicular Tumors: a Clinicopathological Study. *Cancer, N.Y.*, **6**, 427.

DOLL, R. & KINLEIN, L. (1970) Immunosurveillance and Cancer: Epidemiological Evidence. *Br. med. J.*, iv, 420.
DUMONDE, D. C., WOLSTENCROFT, R. A., PANAY, G. S., MATHEW, M., MORLEY, J. & HOWSON, W. T. (1969) " Lymphokines ": Non-antibody Mediators of Cellular Immunity Generated by Lymphocyte Activation. *Nature, Lond.*, **224**, 38.
EVERSON, T. C. & COLE, W. H. (1966) *Spontaneous Regression of Cancer.* Philadelphia: Saunders.
GROSS, R. E., FARBER, S. & MARTIN, L. W. (1959) Neuroblastoma Sympatheticum. A Study and Report of 217 Cases. *Pediatrics, Springfield*, **23**, 1179.
HALL, J. G. (1967) Studies of the Cells in the Afferent and Efferent Lymph of Lymph Nodes Draining the Site of Skin Homografts. *J. exp. Med.*, **125**, 737.
HAMILTON FAIRLEY, G. (1969) Immunity to Malignant Disease in Man. *Br. med. J.*, ii, 467.
HAMLIN, I. M. E. (1968) Possible Host Resistance in Carcinoma of the Breast: A Histological Study. *Br. J. Cancer*, **22**, 383.
HARRIS, J. E. & SINCOVICS, J. G. (1971) *Immunology of Malignant Disease.* St Louis: Mosby.
HELLSTRÖM, I. E., HELLSTRÖM, K. E., PIERCE, G. E. & BILL, A. H. (1968) Demonstration of Cell-bound and Humoral Immunity against Neuroblastoma Cells. *Proc. natn. Acad. Sci. U.S.A.*, **60**, 1231.
KEAST, D. (1970) Immunosurveillance and Cancer. *Lancet*, ii, 710.
LANGLEY, R. (1970) *Practical Statistics.* Newton Abbot: David and Charles.
LEWIS, D. G. (1967) *Statistical Methods in Education.* University of London Press.
LEWIS, M. G., IKONOPISOV, R. L., NAIRN, R. C., PHILLIPS, T. M., HAMILTON FAIRLEY, G., BODENHAM, D. C. & ALEXANDER, P. (1969) Tumor Specific Antibodies in Human Malignant Melanomas and Their Relationship to the Extent of the Disease. *Br. med. J.*, i, 547.
LUKES, R. J. (1964) Prognosis and Relationship of Histologic Features to Clinical Stage. *J. Am. med. Ass.*, **190**, 914.
MACKLER, B. F. (1971) Role of Soluble Lymphocyte Mediators in Malignant Tumour Destruction. *Lancet*, ii, 297.
MARSDEN, H. B. & STEWARD, J. K. (1968) Tumours of the Sympathetic System. *Recent Results in Cancer Res.*, **13**, 131.
MARSHALL, W. H., VALENTINE, F. T. & LAWRENCE, H. S. (1969) Cellular Immunity *in vitro*. Clonal Proliferation of Antigen-stimulated Lymphocytes. *J. exp. Med.*, **130**, 327.
MARTIN, R. F. & BECKWITH, M. D. (1968) Lymphoid Infiltrates in Neuroblastomas, Their Occurrence and Prognostic Significance. *J. pediat. Surg.*, **3**, 161.
MATHÉ, G., AMIEL, J. L., SCHWARZENBERG, L., SCHNEIDER, M., CATTAN, A., SCHLUMBERGER, J. R., HAYAT, M. & DE VASSAL, F. (1969) Active Immunotherapy for Acute Lymphoblastic Leukaemia. *Lancet*, i, 697.
OWEN, D. B. (1962) *Handbook of Statistical Tables.* London: Addison-Wesley.
PIESSENS, W. L. (1970) Evidence for Human Cancer Immunity. *Cancer, N.Y.*, **26**, 1212.
SIEGEL, S. (1956) *Nonparametric Statistics for the Behavioral Sciences.* New York: McGraw-Hill.
STEWART, T. H. M., KLASSEN, D. & CROOK, A. F. (1969) Methotrexate in the Treatment of Malignant Tumours: Evidence for the Possible Participation of Host Defence Mechanisms. *Can. med. Ass. J.*, **101**, 191.
STOWENS, D. (1957) Neuroblastoma and Related Tumors. *Archs Path.*, **63**, 451.
SWAN, H. T. & KNOWELDEN, J. (1971) Prognosis in Hodgkin's Disease Related to the Lymphocyte Count. *Br. J. Haemat.*, **21**, 343.
VANKY, F., STJERNSWÄRD, J. & NILSONNE, U. (1971) Cellular Immunity to Human Sarcoma. *J. natn. Cancer Inst.*, **46**, 1145.
YOON, I. L. (1959) The Eosinophil and Gastrointestinal Carcinoma. *Am. J. Surg.*, **97**, 195.

### APPENDIX

By choosing non-parametric methods of analysis, we avoided the need to assume that our data conformed to the Normal distribution, or indeed any specific distribution. We did encounter the difficulty of tied ranks. This we surmounted by using procedures which were somewhat more complicated than would otherwise have sufficed. These procedures assigned to each rank in a tie the mean of the tied rank values.

(1) In scoring the intensity of lymphocytic infiltration in the tumours we tried to achieve the maximum degree of consistency by examining the sections on 3 separate occasions. The consistency is measurable by Kendall's coefficient of concordance, $W$:

$$W = 12s/[k^2(N^3 - N)];$$

$$s = \sum [R_j - (\sum R_j)/N]^2$$

where $R_j$ is the sum of ranks in a $k \times N$ table, $k$ is the number of sets of rankings (3 in this study) and $N$ is the number of entities ranked (23).

$W$ can take any value between 0 and 1. In a sample as large as $N = 23$ we may use the $\chi^2$ distribution to assess the significance of $W$, because

$$\chi^2 \sim k(N - 1)\,W; \qquad \text{d.f.} = (n - 1)$$

(2) Fisher's exact test enables one to compute directly the probability of any particular grouping of the variables, a, b, c, d in a dichotomous grouping:

Category A

| | I | II | |
|---|---|---|---|
| Category B I[1] | a | b | $n_1$ |
| II[1] | c | d | $n^2$ |
| | $n^3$ | $n^4$ | |

$$N = a + b + c + d$$

$$P = \frac{n_1\,!\,n_2\,!\,n_3\,!\,n_4\,!}{N\,!\,a\,!\,b\,!\,c\,!\,d\,!}$$

(3) The Mann and Whitney $U$ test examines the location of medians. We used the form:

$$U = n_1 n_2 + n_1(n_1 + 1)/2 - R_1$$

where $R_1$ is the sum of the ranks assigned to the ordered group of size $n_1$ and $U$ takes the smaller of its two possible values.

(4) Cooper (1969) gives the following formulation of Spearman's test of rank correlation; it takes account of tied ranks:

$$r_s = \left[\sum_1^n x_i^2 + \sum_1^n y_i^2 - \sum_1^n d_i^2\right] \Big/ 2\left[\sum_1^n x_i^2 \sum_1^n y_i^2\right]^{1/2}$$

where

$$\sum_{i=1}^n x_i^2 = \frac{n}{12}(n^2 - 1) - \sum_{j=1}^{Tx} \frac{t_j}{12}(t_j^2 - 1)$$

and

$$\sum_{i=1}^n y_i^2 = \frac{n}{12}(n^2 - 1) - \sum_{k=1}^{Ty} \frac{t_k}{12}(t_k^2 - 1)$$

In the latter two expressions $t_j$ is the number of $x$-values involved in the $j$th tie, $t_k$ is the number of $y$-values involved in the $k$th tie, $T_x$ is the number of ties in the $x$-values and $T_y$ is the number of ties in the $y$-values.

Spearman's test showed a significant positive correlation between the intensity of lymphocytic infiltration in the tumour and the duration of patient survival. The correlation between the *peripheral blood lymphocytes count* and the intensity of tumour infiltration fell short of significance at the conventional 5% level. The details of this (shorter) calculation are shown here to illustrate the working of Method 4. There is not sufficient space for the numerical details of the other relatively less crucial methods.

*Peripheral blood lymphocyte count vs. intensity of tumour lymphocytic infiltration*

Number of elements $n = 13$ (Table II)

$$\sum_{i=1}^n x_i^2 = \frac{13}{12}(168) - \left\{\begin{array}{l} \frac{2}{12}(3) = 0{\cdot}5 \\ +\frac{2}{12}(3) = 0{\cdot}5 \end{array}\right\}$$

$$= 181{\cdot}0$$

$$\sum_{i=1}^n y_i^2 = \frac{13}{12}(168) - \left\{\begin{array}{l} \frac{2}{12}(3) = 0{\cdot}5 \\ +\frac{4}{12}(15) = 5{\cdot}0 \end{array}\right\}$$

$$= 176{\cdot}5$$

$$\sum_{i=1}^n d_i^2 = 267{\cdot}5$$

$$r_s = \frac{(181{\cdot}0 + 176{\cdot}5 - 267{\cdot}5)}{2\sqrt{181{\cdot}0 \times 176{\cdot}5}} = 0{\cdot}504$$

*Test for significance of* $r_s = 0{\cdot}504$

The null hypothesis that there is no correlation between the variables is tested approximately by a procedure based on the $t$-distribution:

$$t = r_s\,[(n - 2)/(1 - r_s^2)]^{1/2}$$

For samples larger than $n = 10$ $t$ is distributed with $n - 2$ degrees of freedom.

Thus

$$t = 0{\cdot}504\,[11{\cdot}0/0{\cdot}75]^{1/2}$$
$$= 0{\cdot}504\,(3{\cdot}82)$$
$$= 1{\cdot}925$$

$$P\{t > 1{\cdot}925 | \text{d.f.} = 11\} = 0{\cdot}05 < P < 0{\cdot}10$$

Therefore $r_s$ (blood count vs. tumour infiltration) is not significant.

For samples smaller than $n = 10$ the value of

$$\sum_1^n d_i^2 + \sum \frac{t_m}{12}\cdot(m^3 - m)$$

where $t$ is the number of ties involving $m = 2, 3, 4 \ldots$ observations, can be referred to a significance table quoted by Langley (1970) from Owen (1962). This shows the probability of finding a sum of $d_i^2$, plus the corrections for tied values, as large (or as small) as the experimental result, purely by chance in a situation where there is no real correlation between the ranked variables.

(5) The Friedman analysis of variance by ranks

$$\chi_r^2 = [12/\{Nk(k+1)\}] \sum_{j=1}^{k} (R_j)^2 - 3N(k+1)$$

where $N$ is the number of rows, $k$ the number of columns and $R_j$ the sum of the ranks in the $j$th column. The sampling distribution of $\chi_r^2$ is approximated by the $\chi^2$ distribution with $k - 1$ degrees of freedom. The probability of values of $\chi^2$ can thereby be assessed.